# Correlates of Dietary Changes During COVID-19 in Immunosuppressed Individuals and Their Relatives: Alignment with Nutritional Recommendations

**DOI:** 10.3390/healthcare13212799

**Published:** 2025-11-04

**Authors:** Manila Sophasath, Audrey Plante, Chantal Bémeur, Crystèle Hogue, Mélanie Dieudé, Christopher Fernandez-Prada, Sylvain Bédard, Hélène Tessier, Isabelle Doré

**Affiliations:** 1 Centre de Recherche du Centre Hospitalier de l’Université de Montréal (CRCHUM), Montréal, QC H2X 0A9, Canada; manila.sophasath@umontreal.ca (M.S.); audreyplante87@gmail.com (A.P.); chantal.bemeur@umontreal.ca (C.B.); crystele.hogue.chum@ssss.gouv.qc.ca (C.H.); melanie.dieude@umontreal.ca (M.D.); 2 Department of Nutrition, Faculty of Medicine, Université de Montréal, Montréal, QC H3S 2N4, Canada; 3 Canadian Donation and Transplant Research Program (CDTRP), Edmonton, AB T6G 2E1, Canada; sylvainbedard.net@gmail.com; 4 Department of Microbiology, Infectiology and Immunology, Faculty of Medicine, Université de Montréal, Montréal, QC H3C 3J7, Canada; 5 Affaires Médicales et Innovation, Héma-Québec, Montréal, QC H4R 2W7, Canada; smvetmobile@yahoo.com; 6 Department of Pathology and Microbiology, Faculty of Veterinary Medicine, Université de Montréal, Saint-Hyacinthe, QC J2S 8H5, Canada; christopher.fernandez.prada@umontreal.ca; 7 The Research Group on Infectious Diseases in Production Animals (GREMIP), Faculty of Veterinary Medicine, Université de Montréal, Saint-Hyacinthe, QC J2S 2M2, Canada; 8 Centre D’excellence sur le Partenariat Avec les Patients et le Public (CEPPP), Montréal, QC H2X 0A9, Canada; 9 School of Kinesiology and Physical Activity Sciences, Faculty of medicine, Université de Montréal, Montréal, QC H3C 3J7, Canada; 10 Department of Social and Preventive Medicine, School of Public Health, Université de Montréal, Montréal, QC H3C 3J7, Canada

**Keywords:** nutrition, dietary habits, food consumption, eating behaviours, Canada’s food guide, immunosuppressed, physical activity, COVID-19

## Abstract

**Background/Objectives:** The sanitary measures implemented during the COVID-19 pandemic, although essential for limiting the virus propagation, hindered healthy behaviours and negatively affected mental health and quality of life. In immunosuppressed individuals at higher risk of COVID-19 complications, these measures may have influenced adherence to Canada’s Food Guide 2019 (CFG-2019). This study aims to describe whether changes in eating behaviours and food consumption during the COVID-19 pandemic were aligned with CFG-2019 and identify correlates of changes in immunosuppressed individuals and their relatives. **Methods:** A total of 210 participants completed an online questionnaire between May and September 2021. Changes in eating behaviours and food consumption were categorized as no change, change aligned with CFG-2019, or change not aligned. Multinomial logistic regressions examined the association between sociodemographic, lifestyle, clinical, and psychosocial characteristics and changes in eating behaviours and food consumption. **Results:** Participants reporting no change in eating behaviours, change aligned, and change not aligned with CFG-2019 were nearly equivalent (29.4%, 33.8%, and 36.8%, respectively). For food consumption, the proportions were 17.0%, 41.2%, and 41.8%, respectively. Reduced physical activity and elevated anxiety and depressive symptoms were associated with a change in eating behaviours not aligned with CFG-2019. Perceived weight gain and decreased body image satisfaction were associated with a non-aligned change in food consumption. **Conclusions:** Dietary changes, aligned or not with CFG-2019, were observed among immunosuppressed individuals and their relatives during the COVID-19 pandemic. Identifying factors associated with these changes can inform future studies to support healthy eating among vulnerable individuals amidst stressful events such as the COVID-19 pandemic.

## 1. Introduction

The COVID-19 pandemic has led the world into a public health crisis, during which lockdown measures have significantly impacted human life’s activities. Indeed, the confinement policies implemented in most countries, combined with the shutdown of most industries, imposed extended periods of home confinement. For immunosuppressed individuals, the impact of lockdown periods was likely more important, as they were given strict instructions regarding self-isolation and avoidance of social contact, given their increased risk of severe COVID-19 complications [1,2]. Relatives and household members often adopted the same protective behaviours to reduce the risk of transmission within the household. Although essential to protect vulnerable populations, these measures introduced additional barriers to maintain healthy lifestyle behaviours during the pandemic [3,4,5,6] and had a negative impact on the mental status and quality of life of immunosuppressed individuals and their relatives [7].

Immunosuppressed individuals are particularly vulnerable to foodborne infections due to their compromised immune function. They must already adopt specific dietary and behavioural strategies to ensure food safety, such as avoiding high-risk foods and following strict food storage and preparation practices [8,9]. In addition, stressful life events, such as the COVID-19 pandemic, can add another layer of complexity, as they can contribute to lifestyle changes, such as increased intake of processed foods high in refined sugars and saturated fats, and reduced physical activity, that may further impact their health [10,11,12,13,14]. Given that diet and lifestyle are key contributors to both physical and mental health [15,16,17,18], and that the global burden of noncommunicable diseases continues to rise [19,20], it is crucial to understand how pandemic-related changes have affected this already at-risk group. This includes identifying misalignments in food consumption and eating behaviours with current nutritional guidelines and considering them in the development of targeted tools and interventions to better support immunocompromised individuals in future public health crises.

The Canadian nutritional recommendations presented in the latest Canada’s Food Guide (CFG-2019) address both food consumption and eating behaviour components [21]. CFG-2019 provides detailed recommendations for portions and food groups, focusing on a balanced plate that includes nutrient-rich and varied foods. Additionally, advice and practical suggestions on optimizing the environment and context of meals are presented, such as cooking more at home and reducing the consumption of ultra-processed foods. These recommendations all aim to improve overall dietary habits in order to promote global health and optimal outcomes [22].

This study aims to (1) describe whether changes in eating behaviours and food consumption during the COVID-19 pandemic are aligned or not with CFG-2019 recommendations and (2) identify sociodemographic, lifestyle, clinical, and psychosocial correlates of these changes in immunosuppressed individuals and their relatives.

## 2. Materials and Methods

Data were drawn from the COVID-Immuno study that is part of the Projet Laurent, a multidisciplinary research program in Canada initiated and co-developed by patient partners and researchers that aims to evaluate and understand “the risks and benefits of pet ownership in transplanted and immunosuppressed populations” (https://www.projetlaurent.org). In this study, we used cross-sectional data collected during the COVID-19 pandemic, from May to September 2021, among immunosuppressed individuals and their relatives.

Using a convenience sampling strategy, 210 participants were recruited via an invitation advertised on partner organizations’ websites and social media (Canadian Donation and Transplantation Research Program, Kidney Foundation of Canada, Canadian Transplant Association and Cystic Fibrosis Canada). Inclusion criteria for the COVID-Immuno study were (i) aged 15 years and older; (ii) English- or French-speaking; (iii) received an organ transplant, immunosuppressed, a close relative (spouse, children, parent) of a transplant recipient or an immunosuppressed individual, or an organ donor; and (iv) having access to the Internet. Eligible participants who provided online consent were invited to complete a questionnaire using the secure Qualtrics software, version 2021 (Qualtrics, Provo, UT, USA), which implemented automated range restrictions, logic checks, and mandatory responses to reduce entry errors and missing data. As part of the Projet Laurent research program, the COVID-Immuno study received ethics approval from the Research Ethics Committee of the Centre Hospitalier de l’Université de Montréal (15 April 2020; #20.018). All data collection procedures adhered to institutional and public health safety guidelines during the pandemic. The participants completed the questionnaires remotely through online surveys disseminated via partner organizations, ensuring both participant safety and data integrity.

### 2.1. Measures

#### 2.1.1. Eating Behaviours and Food Consumption

In this study, dietary habit is an umbrella term that includes two concepts: eating behaviours and food consumption. Eating behaviour is defined by behavioural recommendations explicitly emphasized in CFG-2019, which encourages individuals to “cook more often,” “eat meals with others,” and “be mindful of eating habits”. Food consumption describes the frequency of consumption of different categories of food. An innovative scoring system was developed to identify whether changes in eating behaviours and food consumption are aligned or not aligned with recommendations from CFG-2019. The alignment score is a simple and transparent analytic tool tailored for this study that allows the classification of the direction and relative magnitude of changes in dietary intake in relation to CFG-2019 recommendations.

To measure change in eating behaviours, the participants were asked to indicate, on a scale of 1 to 5, to what extent each of the following behaviours was more frequent than before the COVID-19 pandemic (1 = much more frequent; 5 = much less frequent): (1) preparing meals, (2) eating in front of a screen, (3) eating with family, and (4) using food delivery services. Changes in food consumption were measured similarly for each of the following categories: (1) fruits and vegetables, (2) legumes, (3) grain products, (4) meat products, (5) dairy, (6) fast foods, (7) instant products, (8) sweets, (9) salty snacks, and (10) alcohol. Then, the behaviour and food consumption components were scored on whether they were aligned or not with the recommendations of CFG-2019 (Table 1). The items in each category (eating behaviours and food consumption) were subsequently summed to obtain an overall score representing whether the participant made changes that were aligned (score < 0) or not aligned (score > 0) with CFG-2019 recommendations.

#### 2.1.2. Anxiety Symptoms

Anxiety symptoms were assessed with the Generalized Anxiety Disorder 7-item scale (GAD7) [23]. The participants reported how often, in the last 7 days, they had been bothered by certain problems (e.g., worrying too much about different things, feeling afraid as if something awful might happen) on a 4-point scale (1 = not at all; 4 = nearly every day). The answers were summed to compute a score that was categorized using validated thresholds (less than 10 = mild anxiety symptoms; more than 9 = moderate to severe anxiety symptoms). The GAD7 is a validated and widely used tool, and its psychometric properties were deemed acceptable to assess anxiety symptoms in a variety of populations [23,24].

#### 2.1.3. Depressive Symptoms

Depressive symptoms were assessed similarly with the 9-item Patient Health Questionnaire (PHQ9) [25]. The participants had to report how often they had been bothered by some problems in the last 7 days (e.g., poor appetite or overeating, feeling down, depressed, or hopeless) on a 4-point scale (1 = not at all; 4 = nearly every day). The answers were summed to a score that was categorized using validated thresholds (less than 10 = mild depressive symptoms; more than 9 = moderate to severe depressive symptoms). The psychometric properties of the PHQ9 were assessed in various populations [24,25].

#### 2.1.4. Resilience

Resilience was assessed with the 7-item Brief Resilience Scale (BRS) [26]. The participants were asked to report how much they agreed or disagreed with several statements (e.g., I tend to bounce back quickly after hard times; I tend to take a long time to get over setbacks in my life) on a scale from 1 to 5 (1 = strongly disagree; 5 = strongly agree). The BRS had acceptable psychometric properties when used in different populations [26,27].

#### 2.1.5. Perception of Body Weight Change

Perception of body weight change was assessed with the following question: Did you notice any changes in your body weight since the beginning of the COVID-19 pandemic in Canada (March 2020)? The response options included (i) I noticed a weight gain, (ii) I noticed a weight loss, and (iii) I did not notice any change. Therefore, the perception of body weight change variable included three categories: weight gain, weight loss, and no change.

#### 2.1.6. Body Image Change

Body image change was assessed by asking the participants: Have you noticed a change in your perception of your body image since the start of the COVID-19 pandemic in Canada (March 2020)? The responses were collected using a 5-point Likert scale (1 = I like my body image a lot more, 2 = I like my body image a little more, 3 = I like my body image no more and no less, 4 = I like my body image a little less, 5 = I like my body image a lot less). Body image was recoded in a three-category variable to compare the participants who liked their body image more (1 and 2), no more or no less (3), and less (4 and 5).

#### 2.1.7. Physical Activity and Sedentary Behaviour

Changes in physical activity and sedentary behaviour were measured using questions developed specifically for this study and adapted from the validated International Physical Activity Questionnaire (IPAQ) [28] to capture behavioural and lifestyle changes occurring during the COVID-19 pandemic, in line with the study objectives. Specifically, the participants were asked to report changes in their frequency of (i) vigorous intensity physical activity (VPA), (ii) moderate intensity physical activity (MPA), and (iii) walking, using the following question: Since the start of the pandemic (compared to before the pandemic), would you say that your [walking frequency] per week… 1 = has increased a lot, 2 = has increased a little, 3 = has not changed, 4 = has decreased a little, or 5 = has decreased a lot. Similar questions were used for moderate and vigorous intensity physical activity. Sedentary behaviour change was assessed using the following question: Since the start of the pandemic (compared to before the pandemic), would you say your number of hours spent sitting, reclining or lying down during awake time on a typical weekday… 1 = has increased a lot… 5 = has decreased a lot. The responses were coded as three-category variables to distinguish between increases (1 and 2), no changes (3), and decreases (4 and 5) in walking, MPA, VPA, and sedentary behaviour.

#### 2.1.8. Clinical and Sociodemographic Characteristics

The participants reported their immunosuppression status according to the following categories: transplanted, immunosuppressed (not transplanted), or donor and relative. Data on sociodemographic variables included age (18–34, 35–54, 55+ years), gender (man, woman, non-binary), employment status (employed (including full and part-time workers and students), unemployed) and household composition (living alone, with a partner, with other family members or friends).

### 2.2. Statistical Analysis

A descriptive analysis of the participant characteristics was conducted. The distribution of missing data across potential correlate variables was then examined. The median (interquartile range; minimum–maximum) percentage of missing values across potential correlates was 3.8% (0.1–6.4; 0–13.7). Individuals with missing values for a specific variable included in a respective model were removed from the analysis. To investigate the association between each potential correlate and changes in eating behaviours and food consumption, a set of two multinomial logistic regression models was conducted, including a univariate, unadjusted model and a multivariable model adjusted for potential confounders. To minimize issues of multiple testing, each set comprised an independent study that addressed a specific hypothesis (one per potential correlate) based on VanderWeele’s Disjunctive Cause Criterion [29]. The significance criterion α was set at *p* ≤ 0.05. The analysis was conducted using R version 4.1.2 (R Core Team 2021).

## 3. Results

A total of 210 participants completed the online questionnaire between May and September 2021. Only one non-binary participant was excluded from the analysis due to gender adjustment. Data on changes in eating behaviours were provided by 201 participants, while 194 participants reported data on food consumption. Participants’ characteristics and missing values for each variable of interest is available in Supplementary Materials (Table S1).

In our sample, 29.4% reported no change in eating behaviours, while 33.8% and 36.8% reported changes aligned and not aligned with CFG-2019 guidelines, respectively. For food consumption, 17.0% reported no change, and a similar proportion of the sample reported changes aligned (41.2%) and not aligned (41.8%) with CFG-2019 guidelines.

Table 2 shows the sociodemographic and clinical characteristics of the total sample, according to the participants’ changes in eating behaviours and food consumption. The majority of participants were women (76.2%), aged 35–54 years (45.7%), employed (69.9%), and an equal proportion reported living with a partner (41.1%) or with other family members or friends (40.7%). The sample included transplant recipients (55.7%), immunosuppressed—but not transplanted—individuals (33.3%) and donors or relatives of a transplanted or immunosuppressed individual (11.0%).

Table 3 presents the results from the multinomial regression unadjusted and adjusted models for potential correlates of changes in eating behaviours. A decrease in walking frequency was associated with an increased odds of a change in eating behaviours not aligned with CFG-2019 recommendations (AOR [95% CI]: 3.10 [1.19; 8.15]). Similarly, a decrease in VPA was associated with an increased odds of a change in eating behaviours not aligned with CFG-2019. However, this association did not remain statistically significant in the adjusted model (AOR [95% CI]: 2.22 [0.96; 5.15]). Interestingly, a decrease in MPA was associated with an increased odds of reporting a change in eating behaviours, both aligned and not aligned with CFG-2019 recommendations. The results show that moderate to severe symptoms of anxiety were associated with an increased odds of reporting a change in eating behaviours not aligned with CFG-2019 (AOR [95% CI]: 2.55 [1.01; 6.42]). Similar results were observed for moderate to severe symptoms of depression (AOR [95% CI]: 2.49 [1.06; 5.84]). The participants with a low resilience level were more likely to report a change in eating behaviours not aligned with CFG-2019 (AOR [95% CI]: 3.28 [1.38; 7.78]).

Table 4 presents the results from the multinomial regression unadjusted and adjusted models for potential correlates of a change in food consumption. The participants who reported a perception of weight gain during the COVID-19 pandemic displayed an increased odds of reporting a change in food consumption not aligned with CFG-2019 (AOR [95% CI]: 4.15 [1.44; 11.88]). The participants who expressed less satisfaction with their body image compared to prior to the COVID-19 pandemic had a higher odds of reporting a change in food consumption not aligned with CFG-2019 (AOR [95% CI]: 4.05 [1.51; 10.88]). These results suggest that the participants who perceived an increase in their body weight or who reported liking their body image less compared to before the COVID-19 pandemic were approximately 4 times more likely to have a change in food consumption not aligned with the recommendations.

## 4. Discussion

This study first aimed to describe reported changes in dietary habits (namely, eating behaviours and food consumption) aligned or not with CFG-2019 guidelines in immunosuppressed individuals and their relatives during the COVID-19 pandemic. Second, this study identified sociodemographic, clinical, behavioural, and psychosocial correlates of these changes. The findings indicate that a majority of immunosuppressed individual and their relatives reported changes in dietary habits in the context of the COVID-19 pandemic. Interestingly, a similar proportion of participants reported changes aligned and not aligned with nutritional recommendations for both eating behaviours and food consumption. Also, distinct correlates of changes in eating behaviours and food consumption were found; while changes in physical activity behaviour (walking, MPA, VPA), symptoms of anxiety and depression, and resilience levels were associated with a change in eating behaviours, changes in body weight perception and body image were associated with a change in food consumption. In our study, gender, age, employment status, household composition, and immunosuppression status were not associated with changes in food consumption or eating behaviours. This finding is partly consistent with previous research, as sociodemographic and clinical factors have generally shown weak or inconsistent associations with dietary changes during the COVID-19 pandemic. Other determinants, such as psychological distress, lifestyle habits, and socioeconomic constraints, have emerged as stronger correlates of changes in dietary habits [30,31,32,33].

Specifically, in regard to eating behaviours, a reduction in physical activity levels, notably, a decrease in walking frequency, as well as in MPA practice and VPA frequency, was associated with an increased odds of a change not aligned with CFG-2019 recommendations. A decrease in MPA was, however, also associated with an increased odds of a change positively aligned with the guidelines. In addition, an association was found between moderate to severe symptoms of anxiety and depression and an increased odds of change in eating behaviours not aligned with CFG-2019. Regarding food consumption, both perception of body weight gain and reduced body image satisfaction were associated with an increased odds of a change not aligned with CFG-2019 recommendations.

Changes in diet and physical activity have been observed in other vulnerable groups during the COVID-19 pandemic, including in older adults, where unfavourable changes in food quantity or quality were observed, as well as decreased physical activity and increased sedentary time [34]. Moreover, the association between physical activity and eating behaviours observed in the current study is consistent with findings from a study among Brazilian adults during the COVID-19 quarantine, where a positive association between physical activity and healthier eating habits was reported [35]. The relationship between physical activity and dietary habits is well documented [36,37,38,39]. It is common for individuals to present with several health risk factors related to lifestyle, such as exhibiting sedentary behaviours and poor dietary habits [40]. The clustering of suboptimal lifestyle habits has been shown to have a synergic effect, negatively impacting health status [41].

We also observed that higher moderate to severe depressive and anxiety symptoms were associated with an increased odds of eating behaviours not aligned with CFG-2019 recommendations, which echo the association between poor dietary habits and mental disorder symptoms that is well reported in the literature [42,43,44]. Similar to our results, many studies observed that the increased levels of anxiety and depressive symptoms in the COVID-19 context led to suboptimal dietary habits, such as high consumption of sweets and fast foods [30,45], especially for sedentary individuals [46].

Our study also suggests that a perception of weight gain is associated with an increased odds of food consumption changes not aligned with CFG-2019 recommendations, including eating more fast food, instant and processed foods, and salty and sweet foods. Those types of foods are widely recognized in the literature as contributors to cardiometabolic risk as well as weight gain across various populations [47,48,49,50]. Moreover, our results suggest an association between lower body image satisfaction and increased changes in food consumption not aligned with CFG-2019, meaning that participants who were dissatisfied with their body image tended to consume poorer nutritional quality foods. Many studies have reported that body image dissatisfaction was associated with (1) “healthier” choices, i.e., higher consumption of fruits and vegetables and avoidance of high-fat foods and breakfast cereals [51,52], (2) restrictive and suboptimal dietary patterns, where participants’ dissatisfaction towards their body image would push them to often skip meals, for instance [52], or (3) higher choices of ultra-processed foods at the store [53], which relates to the results of our cohort. Therefore, the literature shows a contrasting variety of behaviours in response to body image dissatisfaction. Creating nutritional education tools and interventions that convey the importance of healthy eating could more clearly guide individuals to make balanced food choices and could possibly affect their self-esteem and perception of body image in a positive way.

Adhering to the nutritional recommendations outlined in CFG-2019 may help identify and characterize individuals who are prone to making suboptimal dietary changes, thereby informing the development of tailored interventions to improve their eating habits and ultimately support better overall health outcomes. Providing individuals with adapted interventions has proven to be beneficial in reducing health disparities and tends to be more effective and appreciated by individuals [54,55,56]. The relationship between psychological and nutritional behaviours/dietary habits should be considered when designing intervention plans in the context of stressful life events such as the COVID-19 pandemic, especially for immunosuppressed individuals and their relatives, given the stricter preventive isolation measures, which put them and their relatives at higher risk of mental health issues [57]. Addressing the precarious mental health status of individuals experiencing symptoms of depression and anxiety could lead to improvements in dietary habits and promote optimal health outcomes.

The limitations of this study include the cross-sectional design, which does not allow for causal inferences, and self-reported data, which could introduce information misclassification due to recall and social desirability biases. Although a few participants were aged 15–17 years in the COVID-Immuno study’s initial data collection in 2020 [58,59], none were under 18 years old in the current study sample collected in 2021. While the GAD-7, PHQ-9, and BRS were originally validated for adult populations (≥18 years), emerging evidence supports their acceptable psychometric properties among adolescents. Specifically, the GAD-7 shows good internal consistency and factorial validity in adolescent samples [60], and the PHQ-9 demonstrates strong reliability and construct validity in youth populations [61,62]. Given that no participants younger than 18 were included in the present analyses, this limitation does not affect the validity of the reported findings, but it is nonetheless acknowledged for completeness. In addition, the small sample size might restrict the ability to identify statistically significant associations. As suggested in previous research, income level [63] and ethnicity [64] could be associated with dietary patterns. Unfortunately, these variables were not documented in the COVID-Immuno study, which limits our ability to investigate these potential correlates of dietary changes. Moreover, the participants were recruited through partner organizations, which may limit the generalizability of our findings to the broader immunosuppressed population. Although this approach does not necessarily introduce selection bias in terms of internal validity, it may result in a sample that differs in characteristics such as engagement or health literacy compared to non-members of such organizations. Finally, the alignment score developed is an analytic tool tailored for this study, not a validated dietary index. Future research should evaluate its convergent validity by comparing it with established dietary quality indices, which may provide more nuanced assessments of alignment with CFG-2019. Further refinement and validation of such tools could enhance their utility for both research and surveillance purposes. Despite the limitations, to our knowledge, this is the first study to assess the impact of the COVID-19 pandemic on dietary habits among immunosuppressed individuals and their relatives. In fact, many studies investigated the impact of the COVID-19 pandemic on individuals with chronic diseases, but very few targeted transplanted, immunosuppressed individuals and their relatives.

## 5. Conclusions

This study proposed an innovative specific scoring key to characterize dietary habits according to CFG-19 recommendations, which considered not only food consumption but eating behaviours as well. The majority of the immunosuppressed individuals and relatives reported changes in eating behaviours and food consumption, whether aligned or not with CFG-2019, during the COVID-19 pandemic. Our results provide evidence suggesting that a reported decrease in physical activity behaviours, depressive and anxiety symptoms, and low resilience are associated with a higher risk of adhering to eating behaviours mostly not aligned with nutritional recommendations. We also found that perceived weight gain and decreased body image satisfaction relate to changes in food consumption that are not aligned with CFG-2019. A potential area for future research could involve exploring whether encouraging physical activity and providing resources and psychological support to immunosuppressed individuals and their relatives might contribute to facilitating healthier dietary habits. These strategies could be implemented on a broader scale during stressful life events extending beyond the context of the COVID-19 pandemic.

## Figures and Tables

**Table 1:** Scoring key for eating behaviour and food consumption components based on CFG-2019.

	Much More	A Little More	No More, no Less	A Little Less	Much Less
**Eating behaviours**					
Preparing meals	2	1	0	−1	−2
Eating in front of a screen	−2	−1	0	1	2
Eating with family	2	1	0	−1	−2
Using food delivery services	−2	−1	0	1	2
**Food consumption**					
Fruits and vegetables	2	1	0	−1	−2
Legumes	2	1	0	−1	−2
Grain products	2	1	0	−1	−2
Meat products	−2	−1	0	1	2
Dairy	−2	−1	0	1	2
Fast foods	−2	−1	0	1	2
Instant products	−2	−1	0	1	2
Sweets	−2	−1	0	1	2
Salty snacks	−2	−1	0	1	2
Alcohol	−2	−1	0	1	2

**Table 2:** Participant characteristics for the overall sample and by changes in eating behaviours and food consumption during the COVID-19 pandemic; COVID-Immuno Study, 2020–2021, n = 210.

	Overall Sample (n = 210)	Change in Eating Behaviours (n = 201)			Change in Food Consumption (n = 194)		
		No Change (n = 59)	Aligned (n = 68)	Not Aligned (n = 74)	No Change (n = 33)	Aligned (n = 80)	Not Aligned (n = 81)
Gender							
Man	50 (23.8)	14 (23.7)	17 (25.0)	16 (21.6)	6 (18.2)	23 (28.7)	15 (18.5)
Woman	160 (76.2)	45 (76.3)	51 (75.0)	58 (78.4)	27 (81.8)	57 (71.2)	66 (81.5)
Age							
18–34	41 (19.5)	9 (15.3)	9 (13.2)	20 (27.0)	4 (12.1)	11 (13.8)	21 (25.9)
35–54	96 (45.7)	26 (44.1)	34 (50.0)	32 (43.2)	16 (48.5)	31 (38.8)	44 (54.3)
55+	73 (34.8)	24 (40.7)	25 (36.8)	22 (29.7)	13 (39.4)	38 (47.5)	16 (19.8)
Household composition							
Alone	38 (18.2)	12 (20.3)	12 (17.6)	13 (17.8)	6 (18.2)	19 (23.8)	11 (13.8)
W/partner	86 (41.1)	25 (42.4)	28 (41.2)	28 (38.4)	11 (33.3)	36 (45.0)	30 (37.5)
W/other family members or friends	85 (40.7)	22 (37.3)	28 (41.2)	32 (43.8)	16 (48.5)	25 (31.2)	39 (48.8)
Missing	1	-	-	1	-	-	1
Employment status, unemployed	63 (30.1)	20 (33.9)	18 (26.9)	21 (28.4)	12 (36.4)	27 (33.8)	17 (21.2)
Missing	1	-	1	-	-	-	1
Immunosuppression status							
Transplant recipient	117 (55.7)	32 (54.2)	37 (54.4)	40 (54.1)	18 (54.5)	46 (57.5)	41 (50.6)
Immunosuppressed, not transplanted	70 (33.3)	20 (33.9)	21 (30.9)	28 (37.8)	11 (33.3)	23 (28.7)	34 (42.0)
Relative or donor	23 (11.0)	7 (11.9)	10 (14.7)	6 (8.1)	4 (12.1)	11 (13.8)	6 (7.4)

**Table 3:** Results from multinomial logistic regression analysis of correlates of a change in eating behaviours; COVID-Immuno Study, 2020–2021, n = 210.

	Univariable Models (Unadjusted)			Multivariable Models (Adjusted)			
		Aligned	Not Aligned		Aligned	Not Aligned	Covariates Included in Multivariable Model
	n	OR [95% CI]	OR [95% CI]	n	AOR [95% CI]	AOR [95% CI]	
Gender	201			-			No adjustment
Man		Ref	Ref				
Woman		0.93 [0.41; 2.10]	1.13 [0.50; 2.55]		-	-	
Age	201			-			No adjustment
18–34		Ref	Ref				
35–54		1.31 [0.46; 3.76]	0.55 [0.22; 1.42]		-	-	
55+		1.04 [0.35; 3.07]	0.41 [0.16; 1.10]		-	-	
Employment status	200			200			Gender, Age, Immunosuppression Status
Employed or student		Ref	Ref		Ref	Ref	
Unemployed		0.72 [0.33; 1.54]	0.77 [0.37; 1.62]		0.76 [0.29; 1.98]	0.94 [0.37; 2.41]	
Household composition	200			199			Gender, Age, Employment
Alone		Ref	Ref		Ref	Ref	
With partner		1.12 [0.43; 2.94]	1.03 [0.40; 2.68]		1.09 [0.41; 2.91]	1.08 [0.41; 2.84]	
With others		1.27 [0.48; 3.38]	1.34 [0.52; 3.49]		1.16 [0.42; 3.19]	1.27 [0.47; 3.43]	
Immunosuppression status	201			-			No adjustment
Immunosuppressed, not transplanted		Ref	Ref				
Transplanted		1.10 [0.51; 2.39]	0.89 [0.43; 1.87]		-	-	
Relative or donor		1.36 [0.43; 4.27]	0.61 [0.18; 2.10]		-	-	
Sedentary behaviour	201			201			Gender, Age, Employment, Body weight, Body image
No change		Ref	Ref		Ref	Ref	
Decrease		2.53 [0.67; 9.59]	1.34 [0.35; 5.20]		2.87 [0.74; 11.24]	1.30 [0.32; 5.19]	
Increase		2.17 [0.93; 5.05]	1.63 [0.74; 3.56]		1.89 [0.77; 4.64]	1.25 [0.54; 2.91]	
Walking	194			194			Gender, Age, Employment, Body Weight, Body Image
No change		Ref	Ref		Ref	Ref	
Decrease		2.27 [0.84; 6.08]	**3.14 [1.22; 8.10]**		2.32 [0.85; 6.41]	**3.10 [1.19; 8.15]**	
Increase		1.57 [0.68; 3.65]	1.33 [0.58; 3.09]		1.75 [0.71; 4.30]	1.24 [0.51; 3.00]	
MPA	191			191			Gender, Age, Employment, Body Weight, Body Image
No change		Ref	Ref		Ref	Ref	
Decrease		**2.43 [1.03; 5.71]**	**2.68 [1.18; 6.10]**		**2.79 [1.10; 7.01]**	**2.61 [1.09; 6.23]**	
Increase		1.70 [0.68; 4.23]	1.30 [0.52; 3.25]		1.81 [0.71; 4.61]	1.23 [0.49; 3.12]	
VPA	193			193			Gender, Age, Employment, Body Weight, Body Image
No change		Ref	Ref		Ref	Ref	
Decrease		1.88 [0.84; 4.19]	**2.42 [1.10; 5.29]**		1.94 [0.81; 4.66]	2.22 [0.96; 5.15]	
Increase		1.32 [0.49; 3.51]	1.21 [0.45; 3.27]		1.26 [0.45; 3.51]	1.26 [0.44; 3.59]	
Body weight perception	201			201			Gender, Age, Employment, Sedentary Behaviour
No change		Ref	Ref		Ref	Ref	
Weight gain		0.58 [0.23; 1.36]	1.12 [0.46; 2.72]		0.52 [0.23; 1.26]	1.05 [0.42; 2.63]	
Weight loss		0.40 [0.16; 1.03]	1.14 [0.45; 2.87]		0.37 [0.14; 0.95]	1.01 [0.39; 2.60]	
Body image satisfaction	201			201			Gender, Age, Employment, Sedentary Behaviour
No more no less		Ref	Ref		Ref	Ref	
Less		1.15 [0.53; 2.46]	1.87 [0.88; 3.97]		0.99 [0.44; 2.23]	1.84 [0.83; 4.09]	
More		0.90 [0.32; 2.49]	1.19 [0.43; 3.28]		0.84 [0.29; 2.40]	1.02 [0.36; 2.95]	
Anxiety symptoms	192			192			Gender, Age, Employment
Mild		Ref	Ref		Ref	Ref	
Moderate–Severe		0.87 [0.36; 2.80]	**2.56 [1.03; 6.34]**		0.97 [0.34; 2.72]	**2.55 [1.01; 6.42]**	
Depressive symptoms	192			192			Gender, Age, Employment
Mild		Ref	Ref		Ref	Ref	
Moderate–Severe		0.78 [0.33; 2.28]	**2.35 [1.10; 5.94]**		0.87 [0.33; 2.28]	**2.49 [1.06; 5.84]**	
Resilience	180			180			Gender, Age, Employment
Normal		Ref	Ref		Ref	Ref	
High		1.22 [0.45; 3.33]	0.65 [0.20; 2.16]		1.19 [0.43; 3.28]	0.67 [0.20; 2.25]	
Low		1.45 [0.59; 3.59]	**3.30 [1.42; 7.71]**		1.51 [0.61; 3.78]	**3.28 [1.38; 7.78]**	

MPA = moderate intensity physical activity, VPA = vigorous intensity physical activity, OR = odds ratio, AOR = adjusted odds ratio, CI = confidence interval. Bold indicates statistically significant results at *p* < 0.05.

**Table 4:** Results from multinomial logistic regression analysis of correlates of a change in food consumption; COVID-Immuno Study, 2020–2021, n = 210.

	Univariable Models (Unadjusted)			Multivariable Models (Adjusted)			
		Aligned	Not Aligned		Aligned	Not Aligned	Covariates Included in Multivariable Model
	n	OR [95% CI]	OR [95% CI]	n	AOR [95% CI]	AOR [95% CI]	
Gender	194						No adjustment
Man		Ref	Ref				
Woman		0.55 [0.20; 1.51]	0.98 [0.34; 2.79]		-	-	
Age	194						No adjustment
18–34		Ref	Ref				
35–54		0.70 [0.19; 2.57]	0.52 [0.16; 1.80]		-	-	
55+		1.06 [0.29; 3.92]	0.23 [0.06; 0.88]		-	-	
Employment status	193						Gender, Age, Immunosuppression Status
Employed or student		Ref	Ref		Ref	Ref	
Unemployed		0.89 [0.38; 2.08]	0.47 [0.19; 1.15]		0.59 [0.20; 1.77]	0.70 [0.23; 2.16]	
Household composition	193			192			Gender, Age, Employment
Alone		Ref	Ref		Ref	Ref	
With partner		1.03 [0.33; 3.23]	1.49 [0.44; 5.00]		1.16 [0.36; 3.71]	1.63 [0.46; 5.72]	
With others		0.49 [0.16; 1.50]	1.33 [0.42; 4.21]		0.48 [0.15; 1.53]	1.05 [0.31; 3.55]	
Immunosuppression status	194						No adjustment
Immunosuppressed, not transplanted		Ref	Ref				
Transplanted		1.22 [0.50; 3.01]	0.74 [0.12; 2.04]		-	-	
Relative or donor		1.32 [0.34; 5.08]	0.49 [0.31; 1.77]		-	-	
Sedentary behaviour	194			193			Gender, Age, Employment, Body Weight, Body Image
No change		Ref	Ref		Ref	Ref	
Decrease		0.99 [0.27; 3.70]	0.18 [0.03; 1.17]		0.86 [0.22; 3.45]	0.14 [0.02; 0.99]	
Increase		1.16 [0.45; 3.00]	1.50 [0.58; 3.85]		1.17 [0.42; 3.26]	1.09 [0.38; 3.11]	
Walking	188			187			Gender, Age, Employment, Body Weight, Body Image
No change		Ref	Ref		Ref	Ref	
Decrease		0.75 [0.26; 2.17]	0.72 [0.25; 2.03]		0.63 [0.20; 2.02]	0.50 [0.15; 1.63]	
Increase		1.71 [0.60; 4.89]	1.20 [0.42; 3.41]		1.62 [0.52; 4.99]	1.10 [0.35; 3.52]	
MPA	185			184			Gender, Age, Employment, Body Weight, Body Image
No change		Ref	Ref		Ref	Ref	
Decrease		1.16 [0.43; 3.17]	2.20 [0.82; 5.90]		0.92 [0.31; 2.80]	1.10 [0.36; 3.35]	
Increase		1.10 [0.39; 3.12]	0.97 [0.33; 2.90]		0.92 [0.31; 2.77]	0.95 [0.30; 3.03]	
VPA	187			186			Gender, Age, Employment, Body Weight, Body Image
No change		Ref	Ref		Ref	Ref	
Decrease		1.40 [0.57; 3.47]	2.26 [0.91; 5.60]		1.20 [0.45; 3.22]	1.38 [0.51; 3.75]	
Increase		1.80 [0.51; 6.33]	2.29 [0.64; 8.13]		1.55 [0.41; 5.90]	2.02 [0.52; 7.82]	
Body weight perception	194			193			Gender, Age, Employment, Sedentary Behaviour
No change		Ref	Ref		Ref	Ref	
Weight gain		0.71 [0.27; 1.88]	**4.08 [1.51; 11.04]**		0.77 [0.28; 2.11]	**4.15 [1.44; 11.88]**	
Weight loss		1.79 [0.65; 4.94]	2.37 [0.77; 7.34]		1.98 [0.69; 5.69]	2.21 [0.67; 7.33]	
Body image satisfaction	194			193			Gender, Age, Employment, Sedentary Behaviour
No more no less		Ref	Ref		Ref	Ref	
Less		1.48 [0.56; 3.01]	**4.15 [1.59; 10.81]**		1.42 [0.53; 3.81]	**4.05 [1.51; 10.88]**	
More		4.06 [1.02; 16.11]	1.52 [0.33; 7.03]		4.02 [1.01; 16.02]	1.54 [0.33; 7.25]	
Anxiety symptoms	186			185			Gender, Age, Employment
Mild		Ref	Ref		Ref	Ref	
Moderate–Severe		0.93 [0.32; 2.70]	1.09 [0.38; 3.11]		1.03 [0.35; 3.07]	0.98 [0.33; 2.88]	
Depressive symptoms	186			185			Gender, Age, Employment
Mild		Ref	Ref		Ref	Ref	
Moderate–Severe		1.49 [0.50; 4.49]	1.96 [0.67; 5.78]		1.53 [0.50; 4.65]	1.84 [0.61; 5.52]	
Resilience	174			173			Gender, Age, Employment
Normal		Ref	Ref		Ref	Ref	
High		1.28 [0.36; 4.52]	1.06 [0.28; 3.94]		1.38 [0.38; 4.99]	1.13 [0.29; 4.34]	
Low		0.99 [0.38; 2.60]	1.36 [0.53; 3.54]		1.09 [0.41; 2.93]	1.34 [0.50; 3.58]	

MPA = moderate intensity physical activity, VPA = vigorous intensity physical activity, OR = odds ratio, AOR = adjusted odds ratio, CI = confidence interval. Bold indicates statistically significant results at *p* < 0.05.

## Data Availability

The original contributions presented in this study are included in the article/Supplementary Materials. Further inquiries can be directed to the corresponding authors.

## Supplementary Materials

The following supporting information can be downloaded at https://www.mdpi.com/article/10.3390/healthcare13212799/s1, Table S1. Participants’ characteristics and missing values for each variable of interest, COVID-Immuno Study, 2020–2021, n = 210.

## Abbreviations

The following abbreviations are used in this manuscript:

AOR	Adjusted odds ratio
BRS	Seven-item brief resilience scale
CFG-2019	Canada’s Food Guide 2019
CI	Confidence interval
GAD7	Generalized Anxiety Disorder seven-item scale
MPA	Moderate intensity physical activity
OR	Odds ratio
PHQ9	Nine-item Patient Health Questionnaire
VPA	Vigorous intensity physical activity

## References

[1] Banerjee D., Popoola J., Shah S., Ster I.C., Quan V., Phanish M. (2020). COVID-19 Infection in Kidney Transplant Recipients. Kidney Int..

[2] Bhoori S., Rossi R.E., Citterio D., Mazzaferro V. (2020). COVID-19 in Long-Term Liver Transplant Patients: Preliminary Experience from an Italian Transplant Centre in Lombardy. Lancet Gastroenterol. Hepatol..

[3] Ammitzbøll C., Andersen J.B., Vils S.R., Mistegaard C.E., Mikkelsen S., Erikstrup C., Thomsen M.K., Hauge E., Troldborg A. (2022). Isolation, Behavioral Changes, and Low Seroprevalence of SARS–CoV-2 Antibodies in Patients with Systemic Lupus Erythematosus or Rheumatoid Arthritis. Arthritis Care Res..

[4] Lupi D., Binda B., Montali F., Natili A., Lancione L., Chiappori D., Parzanese I., Maccarone D., Pisani F. (2020). Transplant Patients’ Isolation and Social Distancing Because of COVID-19: Analysis of the Resilient Capacities of the Transplant in the Management of the Coronavirus Emergency. Transpl. Proc..

[5] Radtke T., Haile S.R., Dressel H., Benden C. (2021). COVID-19 Pandemic Restrictions Continuously Impact on Physical Activity in Adults with Cystic Fibrosis. PLoS ONE.

[6] Radtke T., Haile S.R., Dressel H., Benden C. (2020). Recommended Shielding against COVID-19 Impacts Physical Activity Levels in Adults with Cystic Fibrosis. J. Cyst. Fibros..

[7] Islam J.Y., Vidot D.C., Havanur A., Camacho-Rivera M. (2021). Preventive Behaviors and Mental Health-Related Symptoms Among Immunocompromised Adults During the COVID-19 Pandemic: An Analysis of the COVID Impact Survey. AIDS Res. Hum. Retroviruses.

[8] Lund B.M., O’Brien S.J. (2011). The occurrence and prevention of foodborne disease in vulnerable people. Foodborne Pathog. Dis..

[9] Health Canada Safe Food Handling for Immunocompromised Individuals. https://www.canada.ca/en/health-canada/services/publications/food-nutrition/safe-food-handling-immunocompromised-individuals.html.

[10] Yau Y.H.C., Potenza M.N. (2013). Stress and Eating Behaviors. Minerva Endocrinol..

[11] Adam T.C., Epel E.S. (2007). Stress, Eating and the Reward System. Physiol. Behav..

[12] Torres S.J., Nowson C.A. (2007). Relationship between Stress, Eating Behavior, and Obesity. Nutrition.

[13] Hannan J., Brooten D., Youngblut J.M., Hildago I., Roche R., Seagrave L. (2015). Physical Activity and Stress in Adult Hispanics. J. Am. Assoc. Nurse Pract..

[14] Huber B.C., Steffen J., Schlichtiger J., Brunner S. (2021). Altered Nutrition Behavior during COVID-19 Pandemic Lockdown in Young Adults. Eur. J. Nutr..

[15] Neuhouser M.L. (2019). The Importance of Healthy Dietary Patterns in Chronic Disease Prevention. Nutr. Res..

[16] Tessier A.-J., Wang F., Korat A.A., Eliassen A.H., Chavarro J., Grodstein F., Li J., Liang L., Willett W.C., Sun Q. (2025). Optimal Dietary Patterns for Healthy Aging. Nat. Med..

[17] Thornton J.S., Frémont P., Khan K., Poirier P., Fowles J., Wells G.D., Frankovich R.J. (2016). Physical Activity Prescription: A Critical Opportunity to Address a Modifiable Risk Factor for the Prevention and Management of Chronic Disease: A Position Statement by the Canadian Academy of Sport and Exercise Medicine. Br. J. Sports Med..

[18] Czosnek L., Lederman O., Cormie P., Zopf E., Stubbs B., Rosenbaum S. (2019). Health Benefits, Safety and Cost of Physical Activity Interventions for Mental Health Conditions: A Meta-Review to Inform Translation Efforts. Ment. Health Phys. Act..

[19] Dietz W.H., Douglas C.E., Brownson R.C. (2016). Chronic Disease Prevention: Tobacco Avoidance, Physical Activity, and Nutrition for a Healthy Start. JAMA.

[20] Byrne D.W., Rolando L.A., Aliyu M.H., McGown P.W., Connor L.R., Awalt B.M., Holmes M.C., Wang L., Yarbrough M.I. (2016). Modifiable Healthy Lifestyle Behaviors: 10-Year Health Outcomes From a Health Promotion Program. Am. J. Prev. Med..

[21] Health Canada Canada’s Food Guide. https://food-guide.canada.ca/en/.

[22] Brassard D., Elvidge Munene L.-A., St-Pierre S., Gonzalez A., Guenther P.M., Jessri M., Vena J., Olstad D.L., Vatanparast H., Prowse R. (2022). Evaluation of the Healthy Eating Food Index (HEFI)-2019 Measuring Adherence to Canada’s Food Guide 2019 Recommendations on Healthy Food Choices. Appl. Physiol. Nutr. Metab..

[23] Spitzer R.L., Kroenke K., Williams J.B.W., Löwe B. (2006). A Brief Measure for Assessing Generalized Anxiety Disorder: The GAD-7. Arch. Intern. Med..

[24] Conway A., Sheridan J., Maddicks-Law J., Fulbrook P., Ski C.F., Thompson D.R., Doering L.V. (2016). Accuracy of Anxiety and Depression Screening Tools in Heart Transplant Recipients. Appld. Nurs. Res..

[25] Kroenke K., Spitzer R.L., Williams J.B.W. (2001). The PHQ-9: Validity of a Brief Depression Severity Measure. J. Gen. Intern. Med..

[26] Smith B.W., Dalen J., Wiggins K., Tooley E., Christopher P., Bernard J. (2008). The Brief Resilience Scale: Assessing the Ability to Bounce Back. Int. J. Behav. Med..

[27] Windle G., Bennett K.M., Noyes J. (2011). A Methodological Review of Resilience Measurement Scales. Health Qual. Life Outcomes.

[28] Craig C.L., Marshall A.L., Sjöström M., Bauman A.E., Booth M.L., Ainsworth B.E., Pratt M., Ekelund U., Yngve A., Sallis J.F. (2003). International physical activity questionnaire: 12-country reliability and validity. Med. Sci. Sports Exerc..

[29] VanderWeele T.J. (2019). Principles of Confounder Selection. Eur. J. Epidemiol..

[30] Van Laren A., Drießen M., Rasa S., Massar K., Ten Hoor G.A. (2023). Nutritional changes during the COVID-19 pandemic: A rapid scoping review on the impact of psychological factors. Int. J. Food Sci. Nutr..

[31] Bonaccio M., Costanzo S., Bracone F., Gialluisi A., Di Castelnuovo A., Ruggiero E., Esposito S., Olivieri M., Persichillo M., Cerletti C. (2022). Psychological distress resulting from the COVID-19 confinement is associated with unhealthy dietary changes in two Italian population-based cohorts. Eur. J. Nutr..

[32] Jimenez Rincon S., Dou N., Murray-Kolb L.E., Hudy K., Mitchell D.C., Li R., Na M. (2022). Daily food insecurity is associated with diet quality, but not energy intake, in winter and during COVID-19, among low-income adults. Nutr. J..

[33] Shimpo M., Akamatsu R., Kojima Y., Yokoyama T., Okuhara T., Chiba T. (2021). Factors Associated with Dietary Change since the Outbreak of COVID-19 in Japan. Nutrients.

[34] Elisabeth A.L., Karlen S.B., Magkos F. (2021). The Effect of COVID-19-related Lockdowns on Diet and Physical Activity in Older Adults: A Systematic Review. Aging Dis..

[35] Christofaro D.G.D., Werneck A.O., Tebar W.R., Lofrano-Prado M.C., Botero J.P., Cucato G.G., Malik N., Correia M.A., Ritti-Dias R.M., Prado W.L. (2021). Physical Activity Is Associated With Improved Eating Habits During the COVID-19 Pandemic. Front. Psychol..

[36] Compernolle S., De Cocker K., Teixeira P.J., Oppert J.-M., Roda C., Mackenbach J.D., Lakerveld J., McKee M., Glonti K., Rutter H. (2016). The Associations between Domain-Specific Sedentary Behaviours and Dietary Habits in European Adults: A Cross-Sectional Analysis of the SPOTLIGHT Survey. BMC Public Health.

[37] Sandri E., Capoferri M., Luciani G., Piredda M. (2025). The Link Between Physical Activity, Nutrition, and Health: A Cross-Sectional Study with Multivariate Analysis in a Young and Predominantly Female Spanish Sample. Nutrients.

[38] Gillman M.W., Pinto B.M., Tennstedt S., Glanz K., Marcus B., Friedman R.H. (2001). Relationships of Physical Activity with Dietary Behaviors among Adults. Prev. Med..

[39] Moradell A., Casajús J.A., Moreno L.A., Vicente-Rodríguez G., Gómez-Cabello A. (2023). Effects of Diet-Exercise Interaction on Human Health across a Lifespan. Nutrients.

[40] Ortiz C., López-Cuadrado T., Rodríguez-Blázquez C., Pastor-Barriuso R., Galán I. (2022). Clustering of Unhealthy Lifestyle Behaviors, Self-Rated Health and Disability. Prev. Med..

[41] Noble N., Paul C., Turon H., Oldmeadow C. (2015). Which Modifiable Health Risk Behaviours Are Related? A Systematic Review of the Clustering of Smoking, Nutrition, Alcohol and Physical Activity (‘SNAP’) Health Risk Factors. Prev. Med..

[42] Selvaraj R., Selvamani T.Y., Zahra A., Malla J., Dhanoa R.K., Venugopal S., Shoukrie S.I., Hamouda R.K., Hamid P. (2022). Association Between Dietary Habits and Depression: A Systematic Review. Cureus.

[43] Stea T.H., Alvsvåg L., Kleppang A.L. (2021). The Association between Dietary Habits, Substance Use, and Mental Distress among Adults in Southern Norway: A Cross-Sectional Study among 28,047 Adults from the General Population. Int. J. Environ. Res. Public Health.

[44] Chen H., Cao Z., Hou Y., Yang H., Wang X., Xu C. (2023). The Associations of Dietary Patterns with Depressive and Anxiety Symptoms: A Prospective Study. BMC Med..

[45] Verly-Miguel M., Lopes C.d.S., Freitas J.V., Garcia M.C., Marques M.C., Paravidino V.B., Sichieri R. (2025). Depression, Anxiety and Change in Eating Habits during the COVID-19 Pandemic in Brazilian University Students. PLoS ONE.

[46] Christofaro D.G.D., Tebar W.R., Silva G.C.R., Lofrano-Prado M.C., Botero J.P., Cucato G.G., Malik N., Hollands K., Correia M.A., Ritti-Dias R.M. (2022). Anxiety Is More Related to Inadequate Eating Habits in Inactive than in Physically Active Adults during COVID-19 Quarantine. Clin. Nutr. ESPEN.

[47] Juul F., Martinez-Steele E., Parekh N., Monteiro C.A., Chang V.W. (2018). Ultra-Processed Food Consumption and Excess Weight among US Adults. Br. J. Nutr..

[48] Cordova R., Kliemann N., Huybrechts I., Rauber F., Vamos E.P., Levy R.B., Wagner K.-H., Viallon V., Casagrande C., Nicolas G. (2021). Consumption of Ultra-Processed Foods Associated with Weight Gain and Obesity in Adults: A Multi-National Cohort Study. Clin. Nutr..

[49] Machado P.P., Steele E.M., Levy R.B., Da Costa Louzada M.L., Rangan A., Woods J., Gill T., Scrinis G., Monteiro C.A. (2020). Ultra-Processed Food Consumption and Obesity in the Australian Adult Population. Nutr. Diabetes.

[50] Beslay M., Srour B., Méjean C., Allès B., Fiolet T., Debras C., Chazelas E., Deschasaux M., Wendeu-Foyet M.G., Hercberg S. (2020). Ultra-Processed Food Intake in Association with BMI Change and Risk of Overweight and Obesity: A Prospective Analysis of the French NutriNet-Santé Cohort. PLoS Med..

[51] Bibiloni M.D.M., Pich J., Pons A., Tur J.A. (2013). Body Image and Eating Patterns among Adolescents. BMC Public Health.

[52] Balluck G., Zaynab Toorabally B., Hosenally M. (2016). Association between Body Image Dissatisfaction and Body Mass Index, Eating Habits and Weight Control Practices among Mauritian Adolescents. Mal. J. Nutr..

[53] Medeiros K.J., Zarbato Longo G., Fiates G.M.R. (2023). Food Choices and Perceptions of Consumers with Body Image Dissatisfaction about the “Healthy Foods” Section of a Supermarket. BFJ.

[54] Campbell M.K., Quintiliani L.M. (2006). Tailored Interventions in Public Health: Where Does Tailoring Fit in Interventions to Reduce Health Disparities?. Am. Behav. Sci..

[55] Ryan P., Lauver D.R. (2002). The Efficacy of Tailored Interventions. J. Nurs. Scholarsh..

[56] Baker R., Camosso-Stefinovic J., Gillies C., Shaw E.J., Cheater F., Flottorp S., Robertson N., Wensing M., Fiander M., Eccles M.P. (2015). Tailored Interventions to Address Determinants of Practice. Cochrane Database Syst. Rev..

[57] Al-Rahimi J.S., Nass N.M., Hassoubah S.A., Wazqar D.Y., Alamoudi S.A. (2021). Levels and Predictors of Fear and Health Anxiety during the Current Outbreak of COVID-19 in Immunocompromised and Chronic Disease Patients in Saudi Arabia: A Cross-Sectional Correlational Study. PLoS ONE.

[58] Zeitoun T., Plante A., Sabiston C.M., Dieudé M., Doré I. (2023). The Association between Change in Lifestyle Behaviors and Mental Health Indicators in Immunosuppressed Individuals during the COVID-19 Pandemic. Int. J. Environ. Res. Public Health.

[59] Plante A., Bedrossian N., Cadotte G., Piché A., Michael F., Bédard S., Tessier H., Fernandez-Prada C., Sabiston C.M., Dieudé M. (2023). Pet ownership and lifestyle behaviours of immunosuppressed individuals and their relatives in the context of COVID-19 pandemic. Prev. Med. Rep..

[60] Casares M.Á., Díez-Gómez A., Pérez-Albéniz A., Lucas-Molina B., Fonseca-Pedrero E. (2024). Screening for anxiety in adolescents: Validation of the Generalized Anxiety Disorder Assessment-7 in a representative sample of adolescents. J. Affect Disord..

[61] Andreas J.B., Brunborg G.S. (2017). Depressive Symptomatology among Norwegian Adolescent Boys and Girls: The Patient Health Questionnaire-9 (PHQ-9) Psychometric Properties and Correlates. Front. Psychol..

[62] Richardson L.P., McCauley E., Grossman D.C., McCarty C.A., Richards J., Russo J.E., Rockhill C., Katon W. (2010). Evaluation of the Patient Health Questionnaire-9 Item for detecting major depression among adolescents. Pediatrics.

[63] McCullough M.L., Chantaprasopsuk S., Islami F., Rees-Punia E., Um C.Y., Wang Y., Leach C.R., Sullivan K.R., Patel A.V. (2022). Association of Socioeconomic and Geographic Factors With Diet Quality in US Adults. JAMA Netw. Open.

[64] Bennett G., Bardon L.A., Gibney E.R. (2022). A Comparison of Dietary Patterns and Factors Influencing Food Choice among Ethnic Groups Living in One Locality: A Systematic Review. Nutrients.

