# Supplementary material for: Correlates of Dietary Changes During COVID-19 in Immunosuppressed Individuals and Their Relatives: Alignment with Nutritional Recommendations
Source: Healthcare (Basel). 2025 Nov 4;13(21):2799. doi: 10.3390/healthcare13212799 (PMC12608984; doi:10.3390/healthcare13212799)
Supplement: Supplementary file 1 [file healthcare-13-02799-s001.zip › healthcare-3859923-supplementary.pdf]

Supplementary Table S1. Participants' characteristics and missing values for each variable of interest, COVID-Immuno Study, 2020-2021, n = 210.

	**n (%)**
Gender	
Man	50 (23.8)
Woman	160 (76.2)
Missing	0 (0)
Age	
18-34	41 (19.5)
35-54	96 (45.7)
55+	73 (34.8)
Missing	0 (0)
Employment status	
Employed or student	146 (69.5)
Unemployed	63 (30.1)
Missing	1 (0.5)
Household composition	
Alone	38 (18.2)
With partner	86 (41.1)
With others	85 (40.7)
Missing	1 (0.5)
Immunosuppression status	
Immunosuppressed, not transplanted	117 (55.7)
Transplanted	70 (33.3)
Relative or donor	23 (11.0)
Missing	0 (0)
Sedentary behaviour	
No change	50 (23.8)
Decrease	19 (9.0)
Increase	141 (67.1)
Missing	0 (0)
Walking	
No change	55 (27.8)
Decrease	59 (29.8)
Increase	84 (42.4)
Missing	12 (5.7)
MPA	
No change	73 37.2)
Decrease	76 (38.8)
Increase	47 (24.0)
Missing	14 (6.7)
VPA	
No change	78 (39.4)
Decrease	84 (42.4)
Increase	36 (18.2)
Missing	12 (5.7)

Body weight perception	
No change	57 (28.2)
Weight gain	84 (41.6)
Weight loss	61 (30.2)
Missing	8 (3.8)
Body image satisfaction	
No more no less	83 (41.1)
Less	88 (43.6)
More	31 (15.3)
Missing	8 (3.8)
Anxiety symptoms	
Mild	156 (80.8)
Moderate-Severe	37 (19.2)
Missing	17 (8.1)
Depressive symptoms	
Mild	149 (77.2)
Moderate-Severe	44 (22.8)
Missing	17 (8.1)
Resilience	
Normal	98 (54.1)
High	25 (13.8)
Low	58 (32.0)
Missing	29 (13.8)
Change in eating behaviour	
No change	59 (29.4)
Aligned	68 (33.8)
Not aligned	74 (36.8)
Missing	9 (4.3)
Change in food consumption	
No change	33 (17.0)
Aligned	80 (41.2)
Not aligned	81 (41.8)
Missing	16 (7.6)
